# FTIR-ATR Spectroscopy and Chemometrics for Varietal Screening of PDO Douro Monovarietal Wines: An Exploratory Feasibility Study

**DOI:** 10.3390/molecules31061004

**Published:** 2026-03-17

**Authors:** Ângela Vieira, Amanda Priscila Silva Nascimento, Maria Zélia Branco, Paula Martins-Lopes, José Eduardo Eiras-Dias, João Brazão, Luís Ferreira, Nelson Machado, Ana Novo Barros

**Affiliations:** 1 Centre for the Research and Technology of Agro-Environmental and Biological Sciences (CITAB), Institute for Innovation, Capacity Building, and Sustainability of Agri-Food Production (Inov4Agro), Universidade de Trás-os-Montes e Alto Douro (UTAD), 5000-801 Vila Real, Portugal; angela.sofia.vieira@gmail.com (Â.V.); mzbranco@sapo.pt (M.Z.B.); plopes@utad.pt (P.M.-L.); lmf@utad.pt (L.F.); 2 Academic Unit of Food Engineering, Federal University of Campina Grande (UFCG), Aprígio Veloso Avenue, 882, Campina Grande 58429-900, PB, Brazil; amandapriscil@yahoo.com.br; 3 SENAI Institute of Technology for Operational Efficiency (IST EO), Federation of Industries of the State of Paraíba (FIEPB), Campina Grande 58429-900, PB, Brazil; 4 National Institute for Agricultural and Veterinary Research (INIAV), 2565-191 Dois Portos, Portugal; eiras.dias@iniav.pt (J.E.E.-D.); joao.brazao@iniav.pt (J.B.); 5 Institute for Systems and Computer Engineering, Technology and Science (INESC TEC), Faculty of Engineering, University of Porto (FEUP), 4200-465 Porto, Portugal; nelson.machado@inesctec.pt; 6 NESC TEC Campus da Faculdade de Engenharia, Universidade do Porto, Rua Dr. Roberto Frias, 4200-465 Porto, Portugal

**Keywords:** FTIR-ATR spectroscopy, chemometrics, wine authentication, varietal screening, Partial Least Squares Discriminant Analysis

## Abstract

The authentication of wines with Protected Designation of Origin (PDO) status is a key requirement for quality assurance, traceability, and consumer trust, particularly in traditional wine-producing regions such as the Douro Demarcated Region (Portugal). Among the certification criteria, the reliable identification of grape varieties remains technically challenging, especially when rapid and non-destructive analytical approaches are required. In this study, Fourier-transform infrared spectroscopy coupled with chemometric analysis was evaluated as a rapid screening approach for the differentiation of monovarietal Douro wines produced under standardized microvinification conditions. Twenty-one monovarietal wines were analyzed using mid-infrared spectra (1800–1000 cm^−1^) and classification models were developed using Partial Least Squares Discriminant Analysis (PLS-DA). The PLS-DA models showed preliminary discriminatory capacity, with apparent error rates of 10.2% for calibration and 19.3% under leave-one-out cross-validation. The results indicate that FTIR-ATR spectroscopy combined with chemometrics captures chemically relevant spectral variability associated with grape varietal differences and shows potential as a rapid exploratory screening approach within PDO traceability frameworks. Although the study is based on a limited number of biological replicates from a single vintage and sub-region, the findings provide a methodological baseline for future multi-vintage and multi-region investigations aimed at consolidating FTIR-based approaches for varietal authentication of Douro wines.

## 1. Introduction

The wine sector represents one of the most relevant agro-industrial activities in Europe, combining strong economic importance with significant cultural, historical, and territorial value. Recent analyses based on global statistical reports reveal that, despite pronounced interannual variability driven by climatic conditions and yield fluctuations, global wine production has shown long-term stabilization with volumes near historical averages; however, in 2024 production reached one of the lowest levels observed in recent decades due to adverse climate events and vineyard contraction. At the same time, international wine trade has exhibited resilience in export value, maintaining its strategic contribution to the global agri-food economy [1,2,3]. Within this context, Portugal remains a consolidated traditional Old World producer with sustained contributions to global wine markets and export networks, supported by long-standing viticultural traditions and integration into European agri-food trade [1].

The Douro Demarcated Region, internationally recognized as the world’s oldest legally demarcated wine region, represents a paradigmatic case of the close relationship between geographical origin, grape varieties, and wine typicity, grounded in a long established legal and institutional framework [4]. The region covers approximately 250,000 hectares, of which about 43,700 hectares are currently under vine cultivation, and is subdivided into three sub regions, Baixo Corgo, Cima Corgo, and Douro Superior, each associated with specific climatic and edaphic conditions that influence grape composition and the resulting wine sensory profiles [5]. The interaction between soil, climate, and grape variety, commonly referred to as terroir, plays a decisive role in shaping wine quality, typicity, and authenticity, thereby reinforcing the need for objective and reliable analytical approaches to support traceability systems and protected designation of origin certification schemes [6].

Ensuring the protection of designation of origin and preventing fraudulent practices are essential for maintaining the integrity and market value of wines holding Protected Designation of Origin status. At the European level, wine production and commercialization are governed by a comprehensive legal framework that establishes rules for quality schemes, labeling, and market organization [7]. In Portugal, the certification, control, and inspection of Douro and Port wines are carried out by the Instituto dos Vinhos do Douro e do Porto, which plays a central role in safeguarding the authenticity and traceability of these protected products [5]. Traditional control approaches based on physicochemical parameters and sensory evaluation remain fundamental within regulatory schemes; however, they are often labor intensive, time consuming, and partially subjective. As a result, there is growing interest in complementary analytical methodologies that enable rapid, objective, and high throughput screening for wine authenticity and fraud detection [8].

In this context, Fourier transform infrared spectroscopy has emerged as a versatile analytical tool for wine analysis, enabling rapid data acquisition, minimal sample preparation, and the simultaneous assessment of multiple chemical components within complex matrices [9,10]. When coupled with multivariate statistical techniques, FTIR spectroscopy has demonstrated strong potential for wine classification according to geographical origin, grape variety, and technological factors, supporting its application as a robust screening approach in authenticity and traceability studies [11]. Recent investigations have further highlighted the capacity of mid infrared spectral data combined with chemometric models to discriminate wines from different production regions, reinforcing the relevance of spectroscopic fingerprinting strategies in origin assessment [12]. Similar spectroscopy based chemometric approaches have been successfully applied to other food matrices, including fats and oils, where FTIR methods have proven effective in classification and authentication tasks, underscoring their broader applicability in food authenticity control systems [12,13].

Within the wine sector, previous studies have emphasized the relevance of spectral preprocessing strategies, including derivative transformations and mathematical treatments, to reduce spectral overlap and enhance discrimination performance among samples in spectroscopic datasets [14]. In parallel, visible and **near-infrared spectroscopy** has been increasingly explored for the assessment of physicochemical quality parameters in grapes and fine wines, demonstrating that **spectroscopy-based approaches** can be applied across multiple **analytical** dimensions of wine and grape characterization, from compositional attributes to **quality-related indices** [15,16,17].

Against this background, the present study investigates the application of FTIR ATR spectroscopy combined with Partial Least Squares Discriminant Analysis for the varietal screening of monovarietal wines from the Douro Demarcated Region. Wines were produced under standardized microvinification conditions in order to minimize technological variability and allow a focused assessment of spectral differences associated with grape variety. Given the limited number of independent bottles per variety and the single vintage design, this work is intended as a methodological feasibility study rather than a fully validated authentication model. The results aim to establish a baseline analytical framework that may support future multi vintage and multi region investigations into FTIR based approaches for varietal authentication of protected designation of origin wines.

## 2. Results and Discussion

### 2.1. Spectral Assignment

Mid infrared spectroscopy is widely applied in wine characterization because the vibrational signatures of major chemical constituents generate reproducible absorption patterns that reflect the chemical composition of the wine matrix [18,19,20]. In particular, the spectral region between 1000 and 1800 cm^−1^, which includes a substantial portion of the fingerprint zone, contains informative absorption features related to organic acids, alcohols, phenolic compounds, and carbohydrate related structures commonly present in wines. This spectral window has been extensively explored in combination with multivariate data analysis for the prediction of quality parameters and for supporting wine classification and authentication tasks, reinforcing the suitability of mid infrared based approaches for comprehensive wine analysis [18,19,20].

**Figure 1:**
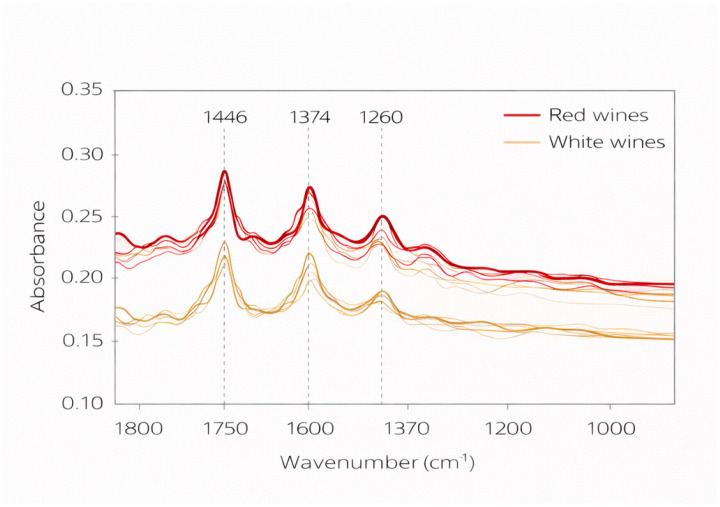
ATR-FTIR absorbance spectra in the 1800–1000 cm^−1^ region of representative red and white monovarietal wines from the Douro Demarcated Region. The spectra illustrate characteristic vibrational bands associated with major wine constituents, including phenolic compounds, organic acids, alcohols, and carbohydrates. The spectra are presented for illustrative purposes only and do not reflect the full extent of varietal variability. The spectral assignments are consistent with previous mid-infrared studies on monovarietal wines and provide a chemical basis for interpreting the spectral variability explored in subsequent chemometric analyses [22,23]. Lighter lines represent individual spectra, while darker lines correspond to the averaged spectra for each wine type.

Figure 1 illustrates representative ATR-FTIR absorbance spectra of red and white wines in the 1800–1000 cm^−1^ region. Although these spectra are shown for visualization purposes only and do not reflect varietal variability, they highlight the main vibrational features observed across the dataset. The most prominent spectral assignments identified in the present study include:

1448–1444 cm^−1^: asymmetric bending of –CH_3_ and deformation of –CH_2_– groups, typically associated with phenolic structures and residual organic acids, has been discussed within the context of mid infrared (MIR) spectral features linked to the fingerprint region of wine spectra, reflecting the presence of organic moieties and compositional variations in wine constituents [18,19].

1376–1373 cm^−1^: symmetric bending vibrations of CH_3_ groups, commonly associated with aliphatic structures and contributions from organic acids and carbohydrate related components present in wine matrices, within the mid infrared fingerprint region [21,22].

1340–1339 cm^−1^: wagging vibrations of CH and CH_2_ groups, frequently linked to polysaccharide related modes and alcohol associated structures in grape and wine matrices investigated by FTIR based approaches combined with multivariate analysis [22,23].

1281–1278 cm^−1^: in plane bending vibrations of OH groups, mainly related to alcohol functional groups and water ethanol interactions typical of hydroalcoholic systems such as wine [23,24].

1207, 1110–1107, and 1068–1062 cm^−1^: C–O stretching vibrations associated with carbohydrates, organic acids, and phenolic related structures, widely reported as informative bands within the fingerprint region of wine mid infrared spectra and frequently exploited in chemometric modeling for quality evaluation and authentication purposes [21,22,24].

**Figure 2:**
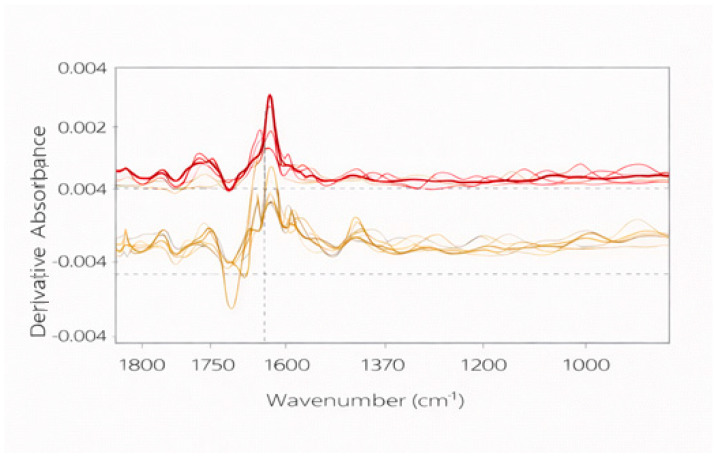
First-derivative ATR-FTIR spectra (1800–1000 cm^−1^) of representative red (red lines) and white (yellow lines) monovarietal wines obtained using a Savitzky–Golay transformation (seven-point smoothing window, second-order polynomial). Derivative spectra are presented to enhance band resolution and facilitate spectral interpretation and were used exclusively for visualization purposes. Lighter lines represent individual spectra, while darker lines correspond to the averaged spectra. Dashed vertical lines indicate characteristic absorption bands used as reference for spectral interpretation, and the dashed horizontal line represents the zero baseline of the derivative absorbance.

To improve spectral visualization and attenuate baseline-related effects, first-derivative spectra were calculated from bottle-level averaged absorbance spectra using a Savitzky–Golay transformation with a seven-point smoothing window and a second-order polynomial. As shown in Figure 2, derivative processing enhanced peak resolution and reduced background contributions while preserving the relative spectral features of the samples [24,25,26].

Derivative spectra were used exclusively for exploratory visualization of spectral features. Although derivative preprocessing is frequently applied in spectroscopic chemometrics to enhance spectral resolution, preliminary exploratory analyses indicated that models built using absorbance spectra provided comparable discrimination while avoiding potential amplification of spectral noise associated with derivative transformations. Therefore, absorbance spectra were retained for the final multivariate modeling.

All chemometric models described in the following sections were developed using the full preprocessed absorbance spectra in the 1000–1800 cm^−1^ range, without manual selection of spectral subregions, in order to minimize bias and ensure methodological transparency.

### 2.2. Exploratory Multivariate Analysis

Given the limited number of biological replicates per grape variety, corresponding to two independent bottles per class, multivariate analysis was conducted on an explora- basis. The primary objective was to evaluate whether mid-infrared spectral profiles contained structured variability consistent with grape varietal differences, rather than to develop a fully validated classification model, an approach commonly adopted in preliminary spectroscopic studies of wine and other complex food matrices [18,23,26,27,28,29,30].

Prior to chemometric modeling, bottle-level averaged spectra were screened for potential outliers using Hotelling’s T^2^ statistics and residual variance analysis, following established practices in multivariate analysis of infrared spectroscopic data applied to wines [10,28,31]. Partial Least Squares Discriminant Analysis was subsequently applied as an exploratory projection method to reduce data dimensionality and visualize dominant patterns within the spectral dataset, as widely reported in FTIR-based authentication and classification studies [18,29,30,31,32,33].

**Figure 3:**
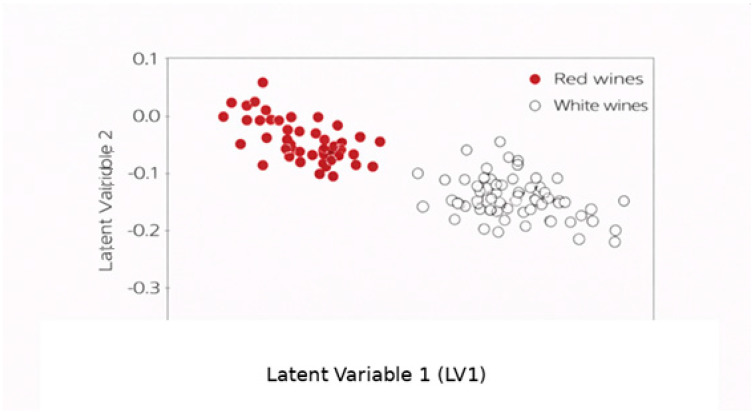
Projection of bottle-level ATR-FTIR spectra onto the first two latent variables (LV1 and LV2) of the PLS-DA model built using the 1000–1800 cm^−1^ spectral range.

Figure 3 shows the projection of bottle-level spectra onto the first two latent variables of the PLS-DA model. The dominant source of variability corresponds to the separation between red and white wines, which is consistent with their well-documented compositional differences, particularly with respect to phenolic content and related chemical features [18,20,29,30]. Within these broader groups, weak tendencies toward grouping were observed for certain grape varieties (e.g., Cabernet Sauvignon, Pinot Noir, Códega do Larinho, and Viosinho), although substantial overlap remained among several samples and no definitive varietal separation can be inferred from the present dataset. This behavior likely reflects chemical similarities between grape varieties, the use of uniform microvinification conditions, and the restricted geographical origin of the samples, as reported in previous exploratory spectroscopic studies of varietal and regional wines [18,29,30,31,33].

These results should be interpreted as an illustration of intrinsic spectral variability captured by ATR-FTIR spectroscopy under controlled production conditions. Rather than defining strict classification boundaries, the analysis highlights the potential of mid-infrared chemometric approaches to support exploratory varietal screening and to inform the design of future studies based on larger, multi-vintage, and multi-region datasets, as commonly recommended for spectroscopic authentication research in complex food matrices [18,23,28,29,30].

Although the present study primarily focuses on the exploratory visualization of spectral variability, the PLS-DA model also provided preliminary classification performance indicators. Exploratory inspection of the derivative spectra suggested that the spectral region between 1495 and 1400 cm^−1^ contributed strongly to the observed discrimination patterns. In this region, apparent classification errors of 10.2% for calibration and 19.3% under leave-one-out cross-validation were obtained. When a more conservative 10-fold cross-validation scheme was applied, slightly higher misclassification levels were observed. Given the limited number of biological replicates per grape variety, these values should be interpreted as preliminary indicators of discriminatory potential rather than validated classification performance.

### 2.3. Practical Implications and Comparison with Alternative Methods

FTIR-ATR spectroscopy offers several practical advantages for wine authentication, particularly in analytical contexts where rapid screening, minimal sample preparation, and low operational cost are key requirements. High-resolution analytical platforms such as nuclear magnetic resonance (NMR), liquid chromatography–mass spectrometry (LC-MS), and gas chromatography–mass spectrometry (GC-MS) provide detailed compositional and metabolomic information and are widely regarded as highly accurate tools for wine characterization and authenticity assessment. However, these techniques typically require more extensive sample preparation, longer analysis times, and higher operational costs. In contrast, FTIR spectroscopy enables rapid spectral acquisition, minimal reagent consumption, and straightforward implementation in routine laboratory workflows. Although FTIR generally provides lower molecular specificity than hyphenated chromatographic or spectrometric techniques, its speed and operational simplicity make it particularly suitable as a high-throughput screening tool prior to confirmatory analyses using more targeted analytical platforms [8,19,25,34]. These advantages have been widely documented for quality control and traceability programs in the wine and broader beverage sectors, positioning FTIR-based approaches as effective first-line analytical tools prior to confirmatory analyses [18,19,20,23,29,30,31,33,35].

In the present study, chemometric analysis of mid-infrared spectra enabled the visualization of systematic spectral variability among monovarietal wines, despite the limited number of biological replicates. Although the dataset does not support the development of validated classification models, the exploratory patterns observed in the PLS-DA projections indicate that MIR spectral profiles capture chemically meaningful information associated with varietal composition. Similar behavior has been reported in previous FTIR-based studies, where exploratory multivariate analysis revealed both discrimination tendencies and partial overlap among wine varieties and geographical origins, reflecting intrinsic compositional similarities and controlled production conditions [18,20,29,30,31,32,33]. These findings support the use of FTIR-based chemometrics as a complementary screening approach within PDO control systems, particularly in scenarios requiring the efficient monitoring of large numbers of samples prior to more targeted or confirmatory analytical procedures.

Additionally, the relatively long storage period between wine production (2012) and spectral analysis (2024) may have introduced chemical changes associated with bottle aging, potentially affecting certain spectral characteristics independently of grape varietal composition. The restricted geographical origin of the samples, the single-vintage experimental design, and the intrinsic chemical similarity among certain Douro grape varieties limit the direct generalization of the findings. Future studies incorporating multi-region and multi-vintage datasets, together with independent validation sets, will be essential to properly assess classification performance and model robustness, as consistently emphasized in the literature on spectroscopic authentication and chemometric modeling [19,23,29,30,31,33]. In this context, the integration of FTIR spectroscopy with complementary analytical approaches, such as targeted phenolic profiling, metabolomics, isotopic analysis, or other hyphenated techniques, has been proposed as an effective strategy to enhance discrimination power, particularly for closely related grape varieties and production systems [20,29,31,33,34,35].

Overall, this study demonstrates that FTIR-ATR spectroscopy combined with chemometric analysis constitutes a scientifically robust and operationally feasible framework for exploratory varietal assessment. Although not intended as a standalone certification tool, this approach can support rapid decision-making, facilitate preliminary screening of extensive sample sets, and contribute to more comprehensive and tiered authentication strategies within PDO regulatory systems, in line with current trends in wine and beverage authenticity research [18,19,20,23,29,30,31,33,34,35].

## 3. Material and Methods

### 3.1. Samples

A total of twenty-one monovarietal wines were analyzed by Fourier-transform infrared (FTIR) spectroscopy using an ATR-FTIR spectrometer (Thermo Fisher Scientific, Waltham, MA, USA).

All wines analyzed in the present study were produced under a standardized microvinification protocol conducted at the National Institute for Agricultural and Veterinary Research (INIAV, I.P., Dois Portos, Torres Vedras, Portugal). Table 1 lists the monovarietal wines included in the spectroscopic dataset, whereas Table 2 summarize the microvinification schemes applied for white and red grape varieties, respectively. The wines analyzed by FTIR correspond to a subset of the microvinified samples described in Table 2. For each grape variety, two independent bottles were selected and considered as biological replicates. For each bottle, three independent sampling sessions were performed, with three consecutive spectra acquired per session, resulting in nine spectra per bottle. These repeated measurements correspond to technical replicates used to evaluate analytical repeatability. For all chemometric analyses, the bottle was defined as the experimental unit.

The wines included both red and white types and were produced from grapes harvested in 2012 in the Douro Demarcated Region (Portugal). With the exception of Gouveio and Malvasia Fina, originating from the Baixo Corgo sub-region, all remaining varieties were sourced from vineyards located in the Cima Corgo sub-region (Table 1). This predominance of a single sub-region may limit the representativeness of the varietal spectral variability observed in the dataset.

Microvinifications were carried out using stainless-steel vessels with capacities of 3, 5, or 10 L, depending on grape availability and experimental design (Table 2). All vinifications were performed under standardized technological conditions including the use of the same facilities, equipment, and oenological products, in order to minimize variability. All bottles were stored under controlled cellar conditions (12–14 °C, absence of light) until analysis. FTIR measurements were performed in 2024. Since the wines were produced from the 2012 harvest and analyzed in 2024, it is acknowledged that long-term bottle storage may have induced chemical changes associated with wine aging, including oxidation reactions, polymerization of phenolic compounds, and esterification processes. Although storage conditions were identical for all samples, these transformations may have influenced certain spectral features and therefore represent a limitation when interpreting the results. Given the limited number of biological replicates per variety, the dataset is intended to support an exploratory feasibility assessment rather than the development of fully validated classification models.

### 3.2. Spectral Acquisition

Before analysis, wine samples were equilibrated to room temperature. For each measurement, 10 µL of wine was deposited directly onto the ATR crystal without additional sample preparation. After each acquisition, the ATR surface was cleaned with ethanol, and a new background spectrum was recorded after every three measurements to ensure baseline stability.

Each bottle was analyzed in three independent sampling sessions, with three consecutive spectra acquired per session, resulting in a total of nine spectra per bottle. All measurements for each bottle were performed within the same analytical session to minimize potential instrumental variability, within the same experimental period under identical conditions. These measurements correspond to technical replicates and were used exclusively to assess the analytical repeatability of the FTIR-ATR procedure. In accordance with the experimental design described in Section 2.1, the bottle was considered the experimental unit for all chemometric analyses.

All spectra were acquired at a spectral resolution of 4 cm^−1^, with 128 scans co-added per measurement to improve the signal-to-noise ratio.

**Figure 4:**
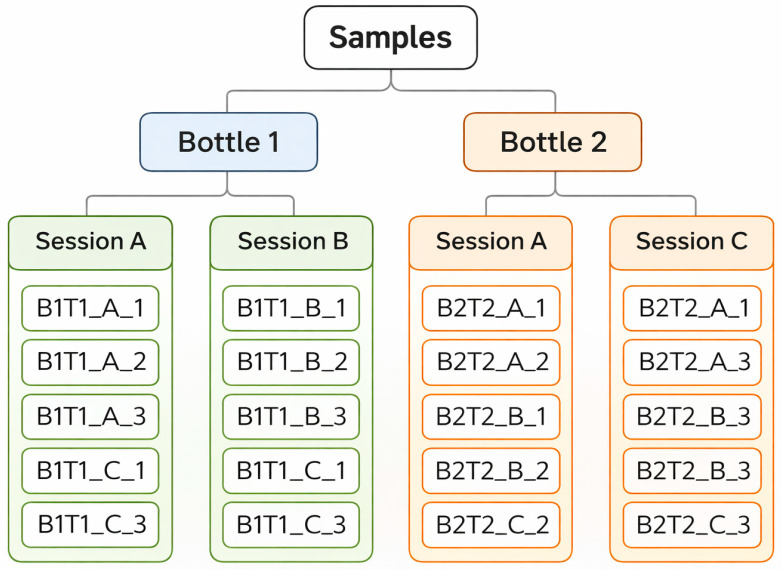
Schematic representation of the acquisition structure of FTIR spectra for each grape Variety.

To clarify the relationship between technical replicates and bottle-level biological replicates used in the chemometric analysis, a schematic representation of the FTIR acquisition structure is shown in Figure 4.

### 3.3. Spectra Treatment

ATR-FTIR spectra were initially processed using the instrument software OMNIC version 9 (Thermo Fisher Scientific, Waltham, MA, USA). The spectral region between 1000 and 1800 cm^−1^ was selected for analysis, as regions outside this interval showed low absorbance and/or increased noise. A fourth-degree polynomial baseline correction was applied with up to 50 iterations, with correction parameters automatically optimized for each spectrum.

After baseline correction, spectra were exported in .csv format and further processed in Origin 9.1 (OriginLab Corporation, Northampton, MA, USA). For each bottle, the nine spectra acquired as technical replicates were inspected to exclude occasional artifacts related to crystal contact, excessive noise, or carryover effects. All spectra meeting these quality criteria were averaged to obtain a single representative spectrum per bottle.

Bottle-level averaged spectra were normalized by dividing absorbance values by the mean spectral intensity. These normalized spectra, corresponding to two independent bottles per grape variety, constituted the dataset used for chemometric modeling. For visualization purposes, first-derivative spectra were computed from the bottle-level averaged spectra using a Savitzky–Golay transformation with a seven-point smoothing window and a second-order polynomial. This preprocessing approach improves spectral resolution by reducing baseline effects and background contributions while preserving relevant spectral features, thereby facilitating the interpretation of overlapping absorption bands and supporting subsequent multivariate analysis [17].

### 3.4. Chemometric Modeling and Classification Strategy

#### Partial Least Squares Discriminant Analysis (PLS-DA)

Chemometric modeling was carried out using Partial Least Squares Discriminant Analysis, a supervised classification approach in which categorical class information is encoded as dummy response variables and regressed against spectral predictors. This strategy allows effective dimensionality reduction while maximizing the covariance between FTIR spectral data and class membership, making it particularly suitable for complex spectroscopic datasets with collinear variables [32,36]. All multivariate analyses were performed using Origin 9.1 (OriginLab Corporation, Northampton, MA, USA).

Prior to model construction, bottle-level averaged spectra were mean-centered. Outlier screening was performed using Hotelling’s T^2^ statistics and residual variance analysis, and no representative bottle-level spectra were excluded.

PLS-DA models were developed using the full preprocessed spectral range (1000–1800 cm^−1^). No manual selection of spectral sub-regions was applied in order to avoid bias and reduce the risk of overfitting. Variable Importance in Projection (VIP) scores were examined exclusively for interpretative purposes.

Given that the experimental unit in this study was the bottle, model validation was carried out using grouped cross-validation, whereby all spectra derived from the same bottle were retained within the same calibration or validation fold. This strategy prevents information leakage and avoids artificially optimistic performance estimates that may arise from technical replicates of the same bottle being split between training and validation sets.

For comparison with previous FTIR-based wine authentication studies involving limited datasets, leave-one-out cross-validation (LOO-CV) was also performed. However, grouped cross-validation was considered the primary and most reliable estimate of model performance.

Classification performance was evaluated in terms of accuracy, sensitivity, and specificity, together with confusion matrices summarizing prediction outcomes. Given the limited number of biological replicates per grape variety, the resulting performance metrics are intended to support a methodological feasibility assessment rather than the validation of a definitive varietal authentication model for PDO certification.

## 4. Conclusions

This study demonstrates that FTIR-ATR spectroscopy captures chemically relevant spectral information that can support exploratory assessment of varietal variability in monovarietal wines. When coupled with chemometric analysis, systematic spectral variability was observed among grape varieties, particularly within the 1000–1495 cm^−1^ region, which is associated with phenolic compounds, organic acids, and carbohydrate-related vibrations. These results reinforce the suitability of mid-infrared spectroscopy as a rapid, non-destructive screening tool for wine characterization.

Although spectral preprocessing strategies, including Savitzky–Golay derivative transformation, enhanced spectral interpretability, the limited number of biological replicates and the absence of external validation preclude the development of fully validated classification models. Accordingly, the findings should be interpreted as exploratory rather than confirmatory. Nevertheless, the consistent patterns observed provide a solid methodological basis for future studies aimed at strengthening FTIR-based varietal discrimination approaches.

Further progress in this field will require expanded datasets encompassing multiple vintages, broader geographical origins, and independent validation sets to enable robust performance assessment. The integration of FTIR spectroscopy with complementary analytical techniques and advanced data-driven approaches may further improve discrimination power, particularly among chemically similar grape varieties.

Overall, FTIR-ATR spectroscopy combined with chemometric tools represents a promising and operationally feasible approach for preliminary varietal screening. With continued methodological refinement and dataset expansion, this strategy may contribute meaningfully to integrated authentication frameworks supporting quality control and origin protection within PDO wine systems.

## Figures and Tables

**Table 1:** Origin of the grapes used in the production of the wine samples analyzed.

Grape Variety	Vineyard/Farm	Sub-Region	Latitude (N)	Longitude (W)
Sousão	Quinta do Seixo	Cima Corgo	41.170	−7.557
Tinta Amarela	Real Companhia Velha	Cima Corgo	41.164	−7.552
Pinot Noir	Quinta de Cidrô	Cima Corgo	41.144	−7.396
Tinta Barroca	Quinta do Seixo	Cima Corgo	41.171	−7.549
Cabernet Sauvignon	Real Companhia Velha	Cima Corgo	41.137	−7.378
Tinta Francisca	Quinta do Seixo	Cima Corgo	41.166	−7.553
Chardonnay	Quinta de Cidrô	Cima Corgo	41.140	−7.385
Tinta Roriz	Quinta do Seixo	Cima Corgo	41.169	−7.549
Moscatel Galego	Quinta de Cidrô	Baixo Corgo	41.257	−7.476
Tinto Cão	Quinta do Seixo	Cima Corgo	41.170	−7.553
Tinta Brasileira	Quinta do Seixo	Cima Corgo	41.168	−7.553
Fernão Pires	Quinta do Casal da Granja	Baixo Corgo	41.257	−7.476
Touriga Franca	Quinta do Seixo	Cima Corgo	41.168	−7.558
Touriga Nacional	Quinta do Seixo	Cima Corgo	41.171	−7.551
Arinto	Quinta de Cidrô	Cima Corgo	41.144	−7.392
Malvasia Fina	Real Companhia Velha	Baixo Corgo	41.258	−7.476
Gouveio	Real Companhia Velha	Baixo Corgo	41.257	−7.476
Riesling	Quinta de Cidrô	Cima Corgo	41.145	−7.396
Merlot	Quinta de Cidrô	Cima Corgo	41.147	−7.398
Cabernet Franc	Quinta de Cidrô	Cima Corgo	41.148	−7.399
Sauvignon Blanc	Quinta de Cidrô	Cima Corgo	41.149	−7.400

**Table 2:** (**a**) Microvinification design and bottle production for white monovarietal wines produced during the INIAV experimental trials. (**b**) Microvinification design and bottle production for red monovarietal wines produced during the INIAV experimental trials.

**(a)**														
**Parameter ↓/Variety →**	**Chardonnay**			**Códega do Larinho**			**Fernão Pires**		**Gouveio**	**Malvasia Fina**		**Moscatel Galego**		**Viosinho**
Microvinification codes	CHARD 1–2			COD.LARIN 1–2			F PIRES 1–3		GOU 1–3	MF 1–3		MG 1–3		VIOS 1–2
Vessel volumes (L) *	10; 5			5			5		5; 3	5; 3		5		5
Total number of bottles	36			24			36		29	29		36		24
**(b)**														
**Parameter ↓/Variety →**	**Alicante B.**	**Aragonez**	**Cab. Sauv.**	**Donzelinho T.**	**Merlot**	**Pinot Noir**	**Rufete**	**Tinta B.**	**Tinta F.**	**Tinto Cão**	**Touriga F.**	**Touriga N.**	**Trincadeira**	**Vinhão**
Microvinification codes	ALIC BOUSC	ARAG	CAB SAUV	DONZ T	MERLOT	PINOT N	RUFET	BARROC	FRANCISC	T CAO	TOUR FRANC	TOUR NAC	TRINCAD	VINHAO
Vessel volumes (L) *	10; 5	10	10; 5; 3	10	10; 5	10; 3	5; 3	10; 5	10; 5	10; 5; 3	10; 5	10	10; 5	10; 5
Total number of bottles	36	48	41	24	36	53	17	36	36	41	36	48	36	36

↓ indicates parameters listed in rows; → indicates grape varieties listed in columns. * Vessel volumes correspond to the capacities of the stainless-steel microvinification vessels used in the experimental trials.

## Data Availability

The data supporting the findings of this study are available from the corresponding author upon reasonable request.
